# Differential effects of SUMO1 and SUMO3 on PKR activation and stability

**DOI:** 10.1038/s41598-018-19683-6

**Published:** 2018-01-19

**Authors:** Ghizlane Maarifi, Faten El Asmi, Mohamed Ali Maroui, Laurent Dianoux, Mounira K. Chelbi-Alix

**Affiliations:** INSERM UMR-S 1124, Université Paris Descartes, 45 rue des Saints-Pères, 75006 Paris, France

## Abstract

Double-stranded RNA (dsRNA)-dependent protein kinase (PKR) is a serine/threonine kinase that exerts its own phosphorylation and the phosphorylation of the α subunit of the protein synthesis initiation factor eIF-2α. PKR was identified as a target of SUMOylation and the triple PKR-SUMO deficient mutant on Lysine residues K60-K150-K440 has reduced PKR activity. We report that SUMO1 and SUMO3 expression exert differential effects on PKR localization, activation and stability. SUMO1 or SUMO3 did not alter the repartition of PKR in the cytoplasm and the nucleus. However, in SUMO3-expressing cells PKR was found more concentrated around the perinuclear membrane and was recruited from small speckles to nuclear dots. Interestingly, SUMO1 expression alone resulted in PKR and eIF-2α activation, whereas SUMO3 reduced PKR and eIF-2α activation upon viral infection or dsRNA transfection. In addition, encephalomyocarditis virus (EMCV) enhanced PKR conjugation to SUMO1 and SUMO3 but only SUMO3 expression promoted caspase-dependent EMCV-induced PKR degradation. Furthermore, the higher EMCV-induced PKR activation by SUMO1 was correlated with an inhibition of EMCV. Importantly SUMO1, by inducing PKR activation in the absence of viral infection, and SUMO3, by counteracting both PKR activation and stability upon viral infection, shed a new light on the differential effects of SUMO-modified PKR.

## Introduction

The small ubiquitin-like modifier (SUMO) family belongs to ubiquitin-like (UBL) proteins^1^. Like other UBL modifiers, SUMOylation involves a cascade of three enzymes: the E1-activating complex SAE1/SAE2, the E2-conjugating enzyme Ubc9 and one of the several E3 ligases. There are five SUMO family members^2–5^, the most studied being SUMO1 and two highly homologous paralogs, SUMO2 and SUMO3, which share 97% sequence identity and cannot be distinguished by currently available antibodies (collectively known as SUMO2/3). SUMO2/3 are expressed at significantly higher levels than SUMO1, they share only 50% sequence identity with SUMO1 and appear to be functionally distinct^3^.

SUMOylation is involved in various cellular processes, such as subcellular localization, protein stability, signal transduction, innate immunity and antiviral defense^2,6–8^. The expression of each SUMO paralog was shown to increase STAT1 SUMOylation and to decrease interferon (IFN)-induced STAT1 activation resulting in an inhibition of IFNγ-induced transcription without affecting that of IFNα^7^. Recently, we reported that the IFN-stimulated gene (ISG) product MxA is conjugated to SUMO at lysine 48^9^, is highly stabilized in cells expressing SUMO and mediates SUMO-induced resistance to vesicular stomatitis virus (VSV)^10^.

Double-stranded (ds) RNA-dependent protein kinase (PKR) is among the ISG products with important biological functions^11,12^. PKR is ubiquitous and constitutively expressed. PKR is induced in an inactive form by IFN and activated by binding to viral dsRNA. This protein is a 68 kDa serine/threonine kinase with two kinase activities, one for its own activation and the other for the phosphorylation of other substrates, the most studied being the α subunit of the protein synthesis initiation factor eIF-2^13,14^. Phosphorylated eIF-2α impairs the activity of the guanine nucleotide exchange factor eIF-2B, resulting in an inhibition of protein synthesis^14^. PKR plays a role in the innate immune response to viral infection and several cellular signal transduction pathways^15^. PKR also appears to function as a tumor suppressor, as expression of inactive mutant forms of the kinase in 3T3 cells converts the cells to a tumorigenic phenotype^16^. In addition, cells expressing human PKR confer partial resistance to encephalomyocarditis virus (EMCV) and accordingly PKR and eIF-2α were phosphorylated^17^.

Through subcellular fractionation, PKR has been found localized mainly in the cytoplasm, with a small fraction in the nucleus^18^. The activities attributed to PKR occur in the cytoplasm whereas the role of nuclear PKR is unclear.

In addition to being phosphorylated, PKR was also identified as a target of ISGylation^19^ and SUMOylation^20^. The authors showed that the triple PKR-SUMO deficient mutant on Lys-60, Lys-150 and Lys-440 has reduced PKR-dsRNA binding, PKR dimerization and eIF-2α phosphorylation^20^. However, whether SUMO expression alters PKR localization, stability or activation is unknown.

In this report, we analyzed the endogenous PKR localization in cells stably overexpressing SUMO1 or SUMO3 and we showed that the SUMO-modified PKR were localized mainly in the nucleus. Although PKR was found equally distributed in the cytoplasm and the nucleus in HeLa-wt, HeLa-SUMO1 and HeLa-SUMO3 expressing cells, PKR staining was found more concentrated around the perinuclear membrane with a recruitment of PKR from small speckles to nuclear dots in SUMO3-expressing cells. In addition, SUMO1 expression activated PKR without virus infection demonstrating a gain-of-function, whereas SUMO3 expression reduced its activation upon viral infection or synthetic dsRNA, poly(I:C), transfection. Furthermore, SUMO3 and not SUMO1 enhanced EMCV-induced PKR degradation. Taken together, our results show that SUMO1 and SUMO3 exert differential effects on PKR localization, protein expression and activation in human cells in response to dsRNA or viral infection.

## Results

### Profile of endogenous PKR

**Figure 1:**
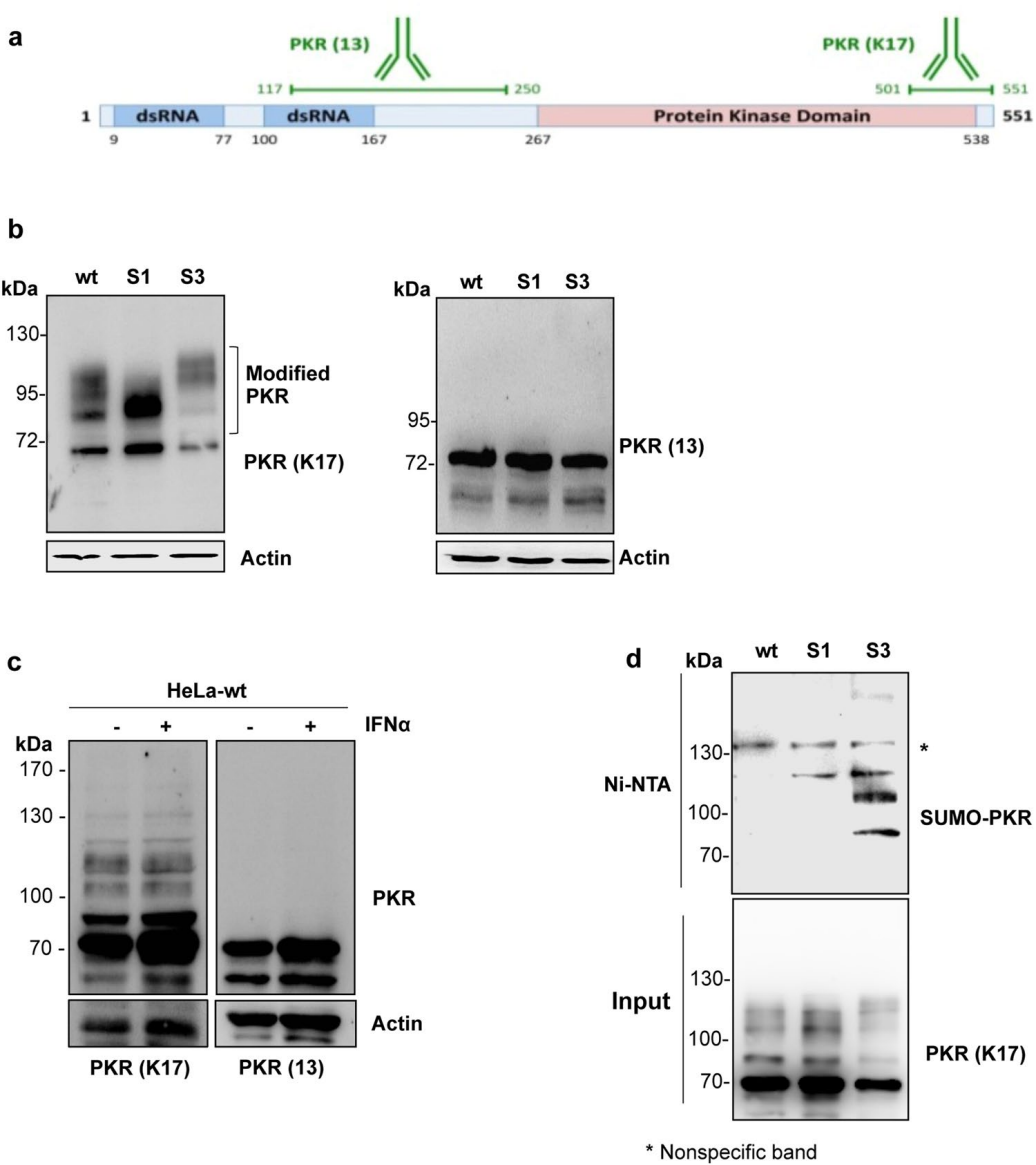
Analysis of anti-PKR antibodies. (**a**) Peptide mapping of rabbit anti-PKR (K17) and mouse monoclonal anti-PKR (13) antibodies is shown. (**b**) Extracts from HeLa-wt, HeLa-SUMO1 or HeLa-SUMO3 cells were analyzed by Western blot using rabbit anti-PKR (K17) (left panel) or mouse monoclonal anti-PKR (13) (right panel) antibodies. (**c**) Extracts from HeLa-wt cells untreated or treated with 1000 units/ml of IFNα for 18 h were analyzed by Western blot using rabbit anti-PKR (K17) or mouse monoclonal anti-PKR (K13) antibodies. (**d**) Extracts from HeLa-wt, HeLa-SUMO1 and HeLa-SUMO3 cells were purified on Ni-NTA-agarose beads. The input and the purified extracts were analyzed by Western blot using anti-PKR (K17) antibody. Uncropped images of blots are shown in Supplementary Figure 1.

First, we analyzed the profile of endogenous PKR in HeLa-wt cells or in HeLa cells stably expressing His-SUMO1 (HeLa-SUMO1) or His-SUMO3 (HeLa-SUMO3). For doing this, two different antibodies were used: rabbit polyclonal anti-PKR (K17) antibody raised against a peptide mapping at the C-terminus of PKR and mouse monoclonal anti-PKR (13) antibody raised against amino acids 117–250 (Fig. 1a). These two antibodies gave radically different profiles in Western blot experiments. PKR (K17) antibody recognized native PKR and also bands of higher molecular weight that correspond to modified forms of PKR. Interestingly, a different profile of modified forms of PKR was observed in HeLa-wt, HeLa-SUMO1 and HeLa-SUMO3 cells (Fig. 1b, left panel). In contrast, analysis of the same cell extracts using anti-PKR (13) antibody revealed only unmodified PKR (Fig. 1b, right panel). Similar results were obtained in HeLa-wt cells untreated or treated with IFNα to enhance PKR expression (Fig. 1c). These results show that only rabbit polyclonal anti-PKR (K17) antibody was able to reveal the modified forms of PKR. Therefore the anti-PKR antibodies used will be indicated in each experiment.

### PKR localization in SUMO-expressing cells

**Figure 2:**
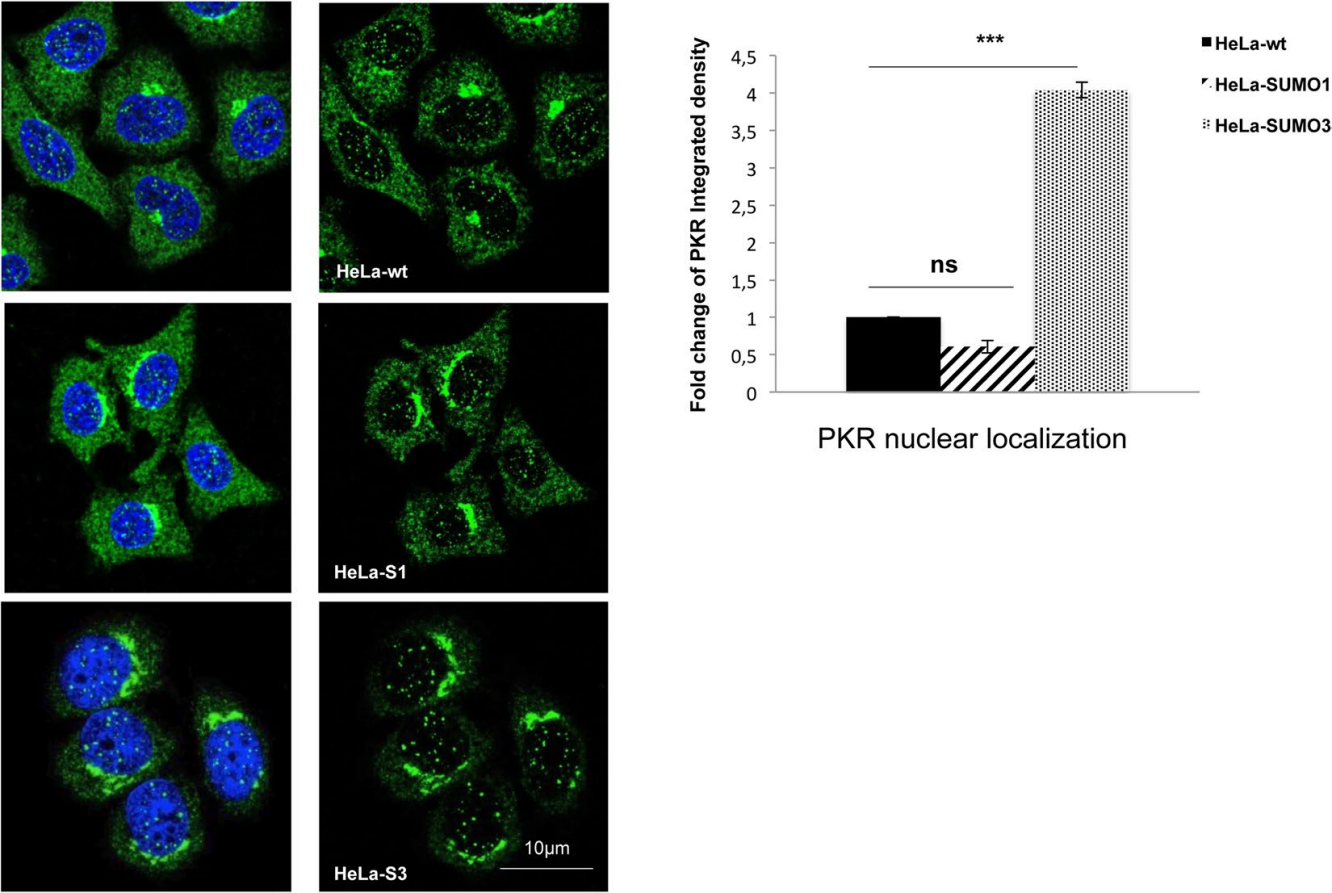
SUMO3 alters PKR localization. Immunofluorescence analysis was performed in HeLa-wt, HeLa-SUMO1 and HeLa-SUMO3 cells for PKR (K17) staining (left panel). Images obtained in wt cells, SUMO1- and SUMO3-expressing cells were quantified using Image-J software (National Institutes of Health). The resulting relative values corresponding to PKR nuclear localization are shown in histograms (n = 15) (right panel). Student *t* test was performed to determine the p value (***p < 0.001), ns: not significant.

SUMO modification has been shown to regulate the subcellular localization or stability of various target proteins^2,10^. Ni-NTA purifications in extracts from His-SUMO1 and His-SUMO3 expressing cells confirm that endogenous PKR is conjugated to SUMO (Fig. 1d). To determine whether the expression of SUMO1 or SUMO3 alters the localization of endogenous PKR, we performed an immunofluorescence analysis in HeLa-wt, HeLa-SUMO1 and HeLa-SUMO3 cells (Fig. 2). In all cells PKR was distributed in the cytoplasm and the nucleus. However, in HeLa-SUMO3 cells, PKR staining was lower in the cytoplasm and was found more concentrated around the perinuclear membrane (Fig. 2). In addition, in the nucleus of wt cells, PKR staining formed many small speckles and SUMO3 expression resulted in the recruitment of PKR to nuclear dots (Fig. 2).

**Figure 3:**
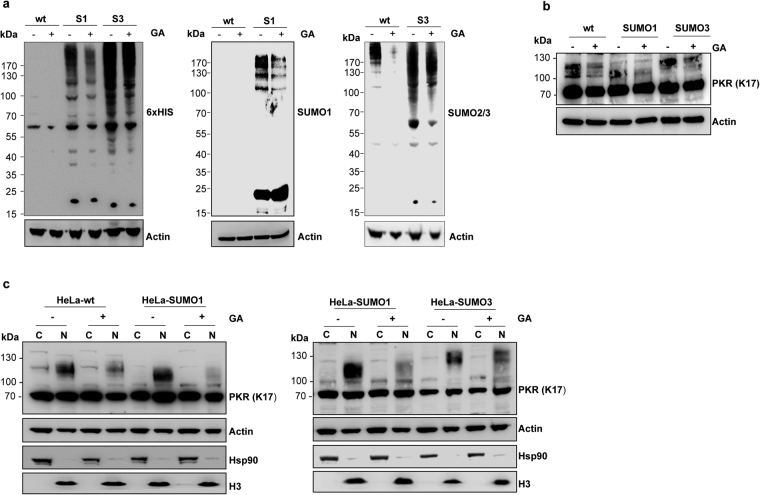
Effect of inhibition of SUMOylation on PKR expression. (**a**) HeLa-wt, HeLa-SUMO1 and HeLa-SUMO3 cells were treated with 100 μM ginkgolic acid (GA) for 6 h. Equal amounts of cell extracts were revealed by Western blot using anti-His (left panel), anti-SUMO1 (middle panel) or anti-SUMO2/3 (right panel) and anti-Actin antibodies. (**b/c**) (**b**) Total cell extracts, (**c**) cytoplasmic (C) and nuclear (N) fractions from cells treated as in a, were analyzed by Western blot for PKR, Hsp90, Histone H3 or Actin. Uncropped images of blots are shown in Supplementary Figure 2.

In a converse experiment, we analyzed the impact of the inhibition of SUMOylation on PKR localization and expression. HeLa-wt, HeLa-SUMO1 or HeLa-SUMO3 cells were untreated or treated with ginkgolic acid (GA), a small molecule inhibitor of SUMO modification, which directly binds E1 and inhibits the formation of the E1-SUMO intermediate^21^, and endogenous PKR expression was analyzed in whole cell extracts, cytoplasmic and nuclear fractions. As expected, GA treatment resulted in a decrease in the level of SUMO1 and SUMO2/3-modified proteins in all extracts (Fig. 3a) and remarkably induced a decrease of PKR-modified forms in all total extracts (Fig. 3b). In addition, cell fractionation revealed that PKR-modified forms were mainly expressed in the nuclear fraction of untreated HeLa-wt, HeLa-SUMO1 and HeLa-SUMO3 cells (Fig. 3c), the levels of which highly decreased upon GA treatment, thus indicating that the SUMO-modified PKR were localized mainly in the nucleus. Furthermore, in untreated HeLa-wt, HeLa-SUMO1 and HeLa-SUMO3 cells, almost the same amount of PKR was found both in the cytoplasm and the nucleus (Fig. 3c). Immunoblotting for Actin showed comparable protein loading and analysis of the distribution of the cytoplasmic marker Hsp90 and the nuclear protein Histone H3 showed that the degree of cross-contamination between the cytoplasmic and the nuclear samples was minimal.

Taken together, these results show that SUMOylated PKR was mainly found in the nucleus and that SUMO1 or SUMO3 expression did not significantly change the distribution of PKR in the cytoplasm and the nucleus. However, the immunofluorescence analysis revealed that SUMO3 expression altered PKR localization in both the cytoplasm and the nucleus, indeed PKR staining was found more concentrated around the perinuclear membrane with the recruitment of PKR from small speckles to nuclear dots.

### Differential effects of SUMO1 and SUMO3 on PKR and eIF-2α phosphorylation

**Figure 4:**
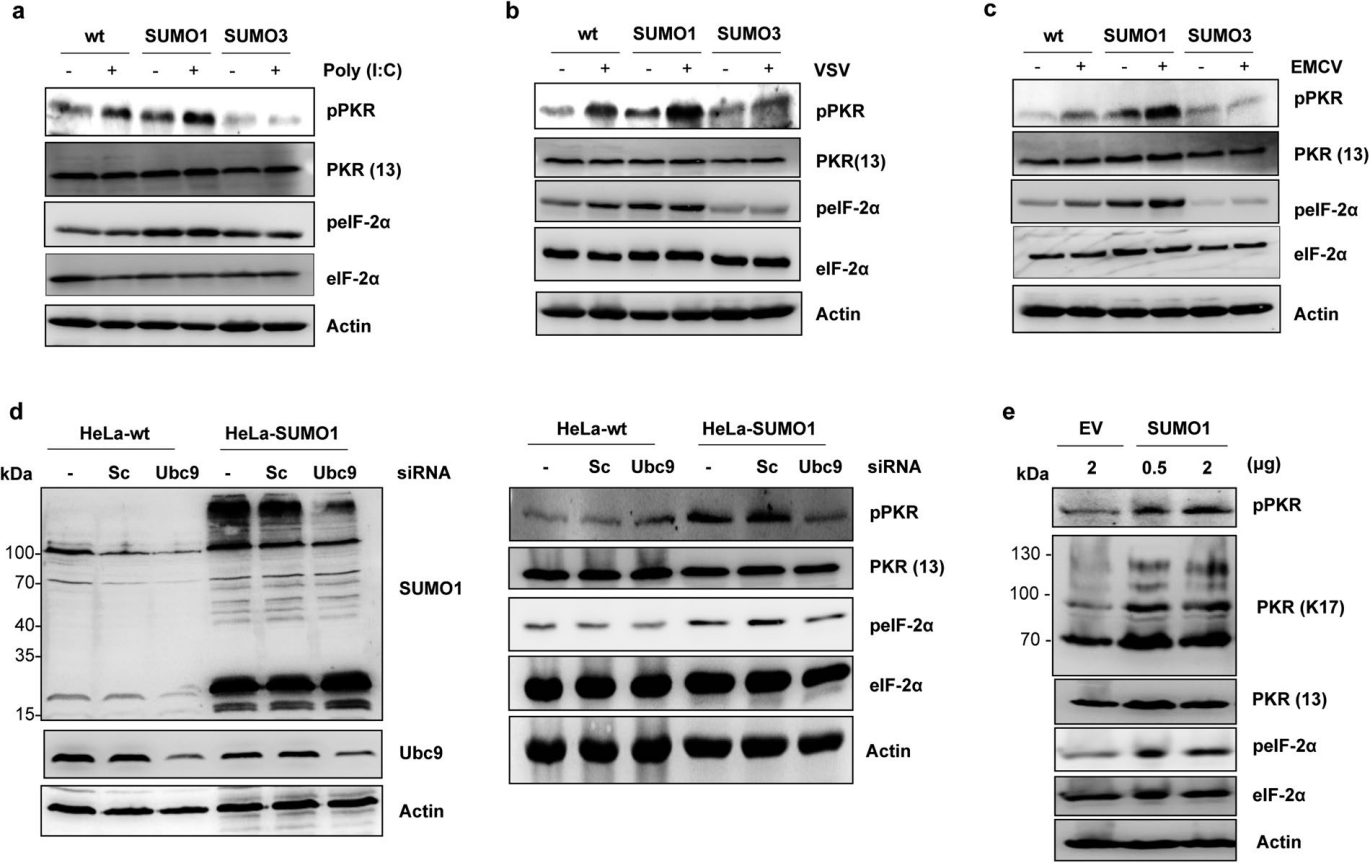
Differential effects of SUMO1 and SUMO3 on PKR and eIF-2α phosphorylation. HeLa-wt, HeLa-SUMO1 and HeLa-SUMO3 cells were transfected with poly(I:C) (**a**), infected with VSV (**b**) or EMCV (**c**) at an MOI of 0.2 for 8 h. Transfected and infected cells were analyzed by Western blot for pPKR and peIF-2α expression. Equal amounts of cell extracts were analyzed with anti-PKR, anti-eIF-2α and anti-Actin antibodies to quantify protein levels. (**d**) HeLa-wt and HeLa-SUMO1 cells were not transfected, transfected with siRNA scramble or siRNA targeting Ubc9. Cell extracts were analyzed by Western blot for Ubc9, SUMO1 (left panel), PKR, pPKR, peIF-2α, eIF-2α and Actin (right panel). (**e**) HeLa-wt cells were not transfected, transfected with 2 μg of empty vector pcDNA3 (EV) or with 0.5 or 2 μg of SUMO1. Cell extracts were analyzed by Western blot for PKR, pPKR, peIF-2α, eIF-2α and Actin. Uncropped images of blots are shown in Supplementary Figure 3.

Next, we investigated the effect of SUMO1 and SUMO3 on PKR and eIF-2α phosphorylation using antibodies directed against phosphorylated PKR and eIF-2α at residue Thr451 and Ser51 respectively. HeLa-wt, HeLa-SUMO1 and HeLa-SUMO3 cells were transfected with poly(I:C) or infected with VSV or EMCV at an MOI of 0.2 for 8 h. Cell extracts analyzed by Western blot revealed that PKR and eIF-2α were phosphorylated in HeLa-wt cells transfected with poly(I:C) (Fig. 4a), infected with VSV (Fig. 4b) or EMCV (Fig. 4c). Remarkably, stable expression of SUMO1 alone resulted in PKR and eIF-2α activation with enhanced phosphorylation of PKR and eIF-2α when cells were transfected with poly(I:C) (Fig. 4a), infected with VSV (Fig. 4b) or EMCV (Fig. 4c). In contrast, in SUMO3-cells, poly(I:C) transfection, VSV or EMCV infection induced a slight and even a non-significant increase of PKR and eIF-2α phosphorylation. It should be noted that the level of PKR protein was not altered upon poly(I:C) transfection (Fig. 4a) and VSV (Fig. 4b) or EMCV (Fig. 4c) infection at an MOI of 0.2.

PKR was found to be activated in cells expressing SUMO1 even in the absence of viral infection or poly(I:C) transfection (Fig. 4a–c). To further demonstrate that PKR activation was dependent on SUMO1, we examined in a reverse experiment the impact of the inhibition of SUMOylation on SUMO1-induced PKR activation. For this purpose, HeLa-wt and HeLa-SUMO1 cells were untreated, transfected with siRNA scramble or siRNA targeting Ubc9. Cell extracts analyzed by Western blot show that specific downregulation of Ubc9 in HeLa-SUMO1 cells was correlated with a decrease of global cellular SUMO1-conjugation (Fig. 4d, left panel) and resulted in a decrease of PKR and eIF-2α phosphorylation (Fig. 4d, right panel). In addition, introducing SUMO1 into HeLa-wt cells by transient transfection activated both PKR and eIF-2α (Fig. 4e). Taken together these results show that SUMO1 expression is able to activate PKR and eIF-2α.

### EMCV enhanced PKR conjugation to SUMO1 and SUMO3 but only SUMO3 promoted EMCV-induced PKR degradation

**Figure 5:**
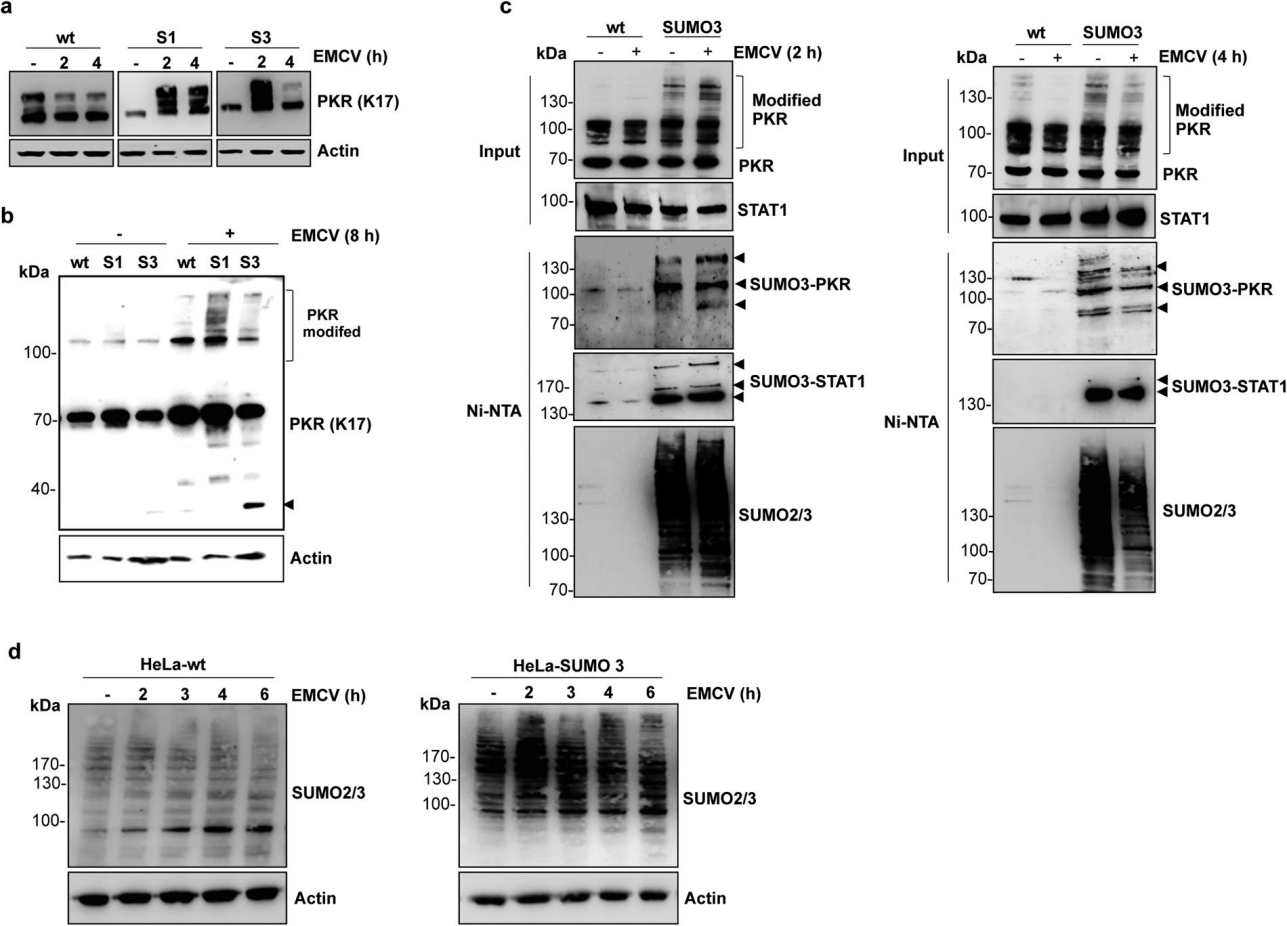
EMCV enhances PKR SUMOylation. ***(*a**) HeLa-wt, HeLa-SUMO1 and HeLa-SUMO3 cells were infected with EMCV at an MOI of 2 for 2 and 4 h and cell extracts were analyzed by Western blot for PKR expression. (**b**) HeLa-wt, HeLa-SUMO1 and HeLa-SUMO3 cells were infected with EMCV at an MOI of 2 for 8 h and cell extracts were analyzed by Western blot using 4–12% gradient gel for PKR and Actin; the arrowhead indicates PKR degradation product. (**c**) HeLa-wt and HeLa-SUMO3 cells were non-infected or infected with EMCV at an MOI of 2 for 2 h (left panel) or 4 h (right panel). Cell extracts from uninfected or infected cells were purified on Ni-NTA-agarose beads. The inputs and the purified extracts were analyzed by Western blot using anti-PKR (K17), anti-STAT1 and anti-SUMO2/3 antibodies; the arrowheads indicate PKR and STAT1 SUMOylated forms. (**d**) HeLa-wt and HeLa-SUMO3 cells were infected with EMCV at an MOI of 2 for different times and cell extracts were analyzed by Western blot using 4–12% gradient gel for SUMO2/3 and Actin. Uncropped images of blots are shown in Supplementary Figure 4.

Previously, it was shown that the decrease of PKR protein is dependent on the multiplicity of EMCV infection^22^. PKR protein downregulation is observed at an MOI of 10 and is not significant at lower MOIs. In order to determine if SUMO is implicated in this process, we analyzed whether EMCV infection alters PKR SUMOylation. HeLa-wt, HeLa-SUMO1 and HeLa-SUMO3 cells were infected with EMCV at an MOI of 2 for 2 or 4 h (Fig. 5a). Compared to HeLa-wt cells, an enhancement of PKR-SUMO1 modified forms was revealed 2 h post-EMCV infection and was maintained 4 h post-infection. In contrast, in HeLa-SUMO3 cells, an enhancement of PKR-modified forms was observed 2 h post-infection, followed by their disappearance at 4 h post-infection (Fig. 5a). To further confirm this result, cells were infected with EMCV at an MOI of 2 for an extended period of 8 h and their extracts were analyzed by Western blot for PKR protein expression (Fig. 5b). PKR was found to be SUMOylated in infected HeLa-wt and HeLa-SUMO1 cells with enhanced PKR-modified forms in SUMO1 cells. In contrast, compared to HeLa-SUMO1, the PKR-modified forms were reduced in HeLa-SUMO3 cells with an appearance of a PKR product migrating at around 37 kDa (Fig. 5b), suggesting that SUMO3 expression promoted EMCV-induced PKR downregulation.

To demonstrate that EMCV enhanced PKR conjugation to SUMO3 2 h post-infection and decreased PKR-SUMO3 modified forms 4 h post-infection, we performed Ni-NTA purifications in extracts from His-SUMO3 expressing cells uninfected and infected with EMCV for 2 h (Fig. 5c, left panel) or 4 h (Fig. 5c, right panel). In the inputs, the unmodified form of PKR migrated at the lowest molecular weight (around 70 kDa), followed by the higher molecular modified species. PKR was found conjugated to SUMO3 with an increase in PKR SUMOylation 2 h post EMCV infection (Fig. 5c, left panel) and the catabolism of the SUMO3-modified PKR 4 h post EMCV infection (Fig. 5c, right panel).

In order to know whether EMCV altered global SUMO2/3 modification, HeLa-wt and HeLa-SUMO3-cells were infected at different times with EMCV at an MOI 2 (Fig. 5d). This experiment revealed that SUMO2/3-modified proteins increased 2 h post-infection and decreased later (Fig. 5d). Although EMCV modulated global SUMO2/3 modification, its effect on PKR 2 h and 4 h post-infection seems to be specific since EMCV did not alter the modification of STAT1 (Fig. 5c) that was shown previously to be SUMOylated^7,23^.

Taken together, these results show that early post-infection, EMCV enhanced PKR-SUMO1 and PKR-SUMO3 modified forms that were decreased later only in SUMO3-expressing cells.

**Figure 6:**
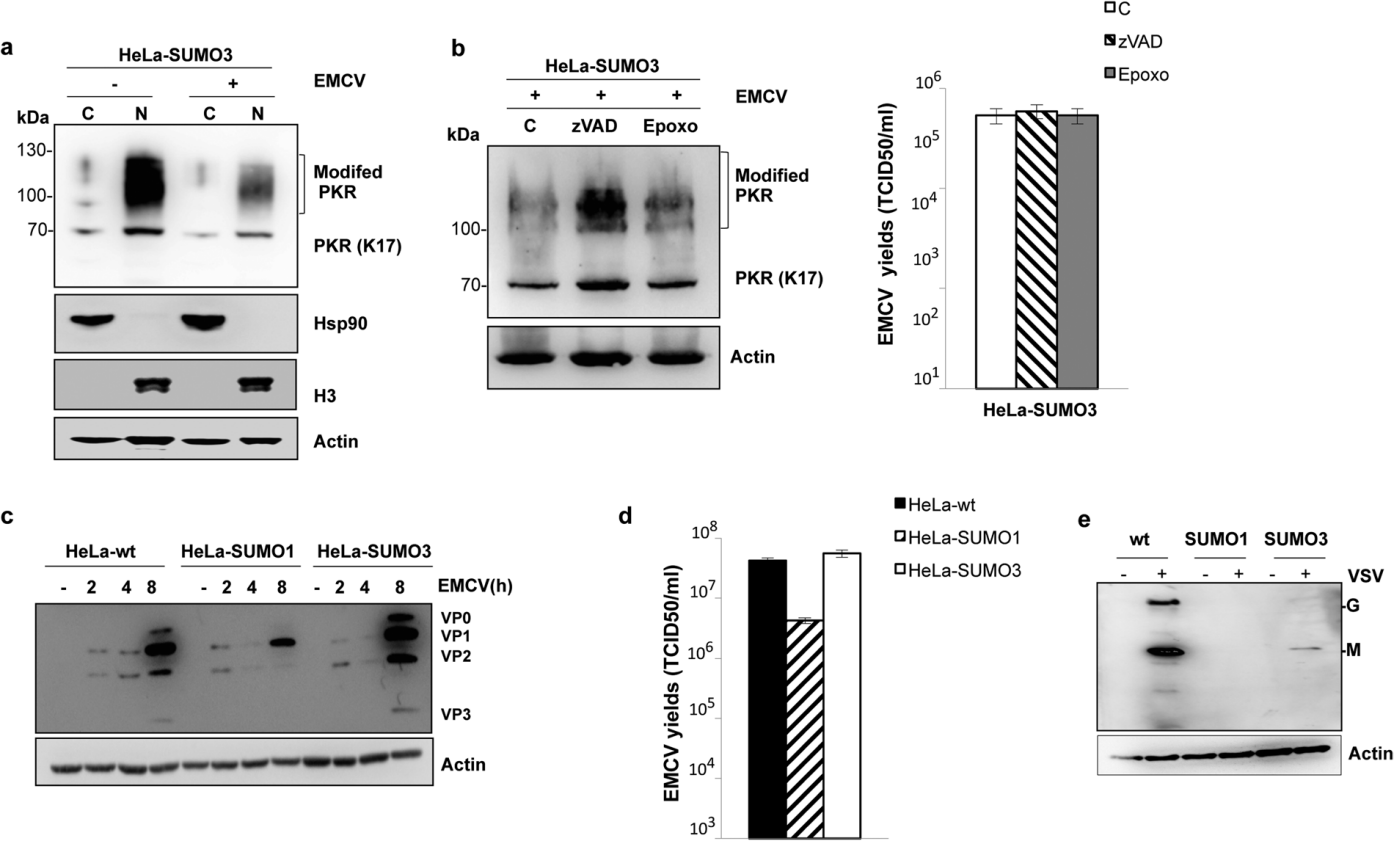
SUMO3 promotes EMCV-induced a caspase-dependent PKR degradation. (**a**) HeLa-SUMO3 cells were non- infected or infected with EMCV at an MOI of 2 for 4 h. Cytoplasmic (C) and nuclear (N) fractions of EMCV-infected HeLa-SUMO3 cells were analyzed by Western blot for PKR, Hsp90, Histone H3 and Actin. (**b**) HeLa-SUMO3 cells were infected with EMCV at an MOI of 2 for 4 h in the absence or presence of zVAD or epoxomicin (Epoxo), their extracts were analyzed by Western blot for PKR and Actin (left panel) and their supernatants were used for the determination of virus titers (right panel) by measuring the 50% tissue culture infective dose (TCID_50_); mean values and standard deviations of three independent experiments are shown. (**c**/**d**) SUMO1 confers partial resistance to EMCV. HeLa-wt, HeLa-SUMO1 and HeLa-SUMO3 cells were infected with EMCV at an MOI of 2 at different times. (**c**) Total cell extracts were analyzed by Western blot for EMCV proteins and Actin and (**d**) the supernatants of infected HeLa-wt, HeLa-SUMO1 and HeLa-SUMO3 cells for 8 h were used for the determination of virus titers by measuring the TCID_50_; mean values and standard deviations of three independent experiments are shown. (**e**) Equal amount of extracts from Fig. 4b of HeLa-wt, HeLa-SUMO1 and HeLa-SUMO3 non-infected or infected with VSV for 8 h were analyzed by Western blot for VSV proteins and Actin. Uncropped images of blots are shown in Supplementary Figure 5.

Next, we analyzed by Western blot PKR expression in cytoplasmic and nuclear fractions of HeLa-SUMO3 cells non-infected or infected with EMCV at an MOI of 2 for 4 h. As seen in Fig. 6a, EMCV infection of HeLa-SUMO3 cells resulted in a decrease in the cytoplasm and the nucleus of the expression of the SUMO-modified and non-modified PKR.

To evaluate whether the proteasome or the caspase might be implicated in the EMCV-induced PKR downregulation, HeLa-SUMO3 cells were infected with EMCV in the presence of the proteasome inhibitor epoxomicin or the caspase inhibitor zVAD (Fig. 6b). The decrease of PKR in EMCV-infected SUMO3 cells was abolished upon treatment with zVAD but not with epoxomicin demonstrating that PKR was degraded *via* the caspase pathway (Fig. 6b, left panel). The concentration used of zVAD^24^ and epoxomicin^25^ did not affect EMCV replication (Fig. 6b, right panel). Also, it should be noted that PKR stabilization after zVAD treatment did not have an impact on EMCV replication in SUMO3-expressing cells because PKR and eIF2 were not activated upon EMCV infection (Fig. 4c).

Taken together, these results show that SUMO3 promoted EMCV-induced caspase-dependent PKR degradation.

### SUMO and EMCV replication

It has been reported that PKR overexpression do not inhibit VSV but confers a partial inhibition to EMCV infection at a low MOI of 0.1^17^. In order to know whether overexpression of SUMO alters EMCV replication, the extracts from HeLa-wt, HeLa-SUMO1 and HeLa-SUMO3 cells infected with EMCV at different times at an MOI of 2, were analyzed by Western blot for the expression of viral proteins (Fig. 6c) and the supernatants were used for the determination of the virus yield (Fig. 6d). Compared to infected HeLa-wt cells, EMCV protein expression (Fig. 6c) and viral titers (1 log reduction) (Fig. 6d) were inhibited in HeLa-SUMO1 cells but were slightly increased in HeLa-SUMO3 cells. In contrast, SUMO1 and SUMO3 expression inhibited VSV protein expression (Fig. 6e), and we reported recently that the anti-VSV effect of SUMO1 and SUMO3 is mediated by MxA protein^10^. Taken together, our results show that the enhanced PKR activation by SUMO1 was correlated with an inhibition of both EMCV protein synthesis and EMCV replication.

## Discussion

We report here that the expression of SUMO1 or SUMO3 did not change the repartition of PKR in the cytoplasm and the nucleus when compared to HeLa-wt cells. However, SUMO3 expression altered PKR localization both in the cytoplasm and the nucleus, indeed PKR staining was found more concentrated around the perinuclear membrane with a recruitment of PKR from small speckles to nuclear dots. In addition, the expression of SUMO1 enhanced the phosphorylation of PKR and eIF-2α in poly(I:C)-transfected cells and in virus-infected cells when compared to wt cells. This is in line with the fact that SUMO1 increases the efficiency of PKR to phosphorylate eIF-2α *in vitro*^20^. Importantly, we found that the expression of SUMO1 was able to activate PKR and eIF-2α in the absence of viral infection suggesting a novel mechanism for PKR activation. It is interesting to note that PKR ISGylation at K69 and K159 by ISG15, another ubiquitin-like modifier, results in PKR and eIF-2α phosphorylation in the absence of viral infection^19^.

In addition, we show that overexpression of SUMO3 in HeLa cells induced PKR SUMOylation, reduced its phosphorylation and the phosphorylation of eIF-2α upon transfection with poly(I:C) and infection with EMCV or VSV at an MOI of 0.2 without affecting the PKR protein level.

It has been previously reported that overexpression of human PKR confers partial resistance to EMCV when cells are infected at low MOIs^17^. Indeed, we report here that the enhanced EMCV-induced PKR and eIF-2α phosphorylation in SUMO1-expressing cells is correlated with an inhibition of both EMCV protein expression and viral titers. In contrast, a slight increase in EMCV production was seen in SUMO3 expressing cells.

It should be noted that SUMO1 and SUMO3 confer resistance to VSV through MxA stabilization^10^ and that MxA depletion in SUMO-expressing cells abrogates the anti-VSV effect of SUMO. Therefore even though PKR activation was altered by SUMO, this has no major consequence on VSV production.

The localization of PKR may vary depending on the viral infection. For example, infection with Human Cytomegalovirus results in the accumulation of PKR in the nucleus^26^. Also, it was hypothesized that the presence of PKR in the nucleus either results in or is a consequence of underphosphorylation of PKR when compared to the cytoplasmic forms of the kinase^18^. Based on our findings, we suggest that SUMO3 conjugation to PKR reduces its activation and PKR-mediated phosphorylation of eIF-2α upon poly(I:C) transfection or viral infections.

In addition, some viruses have developed strategies to alter PKR protein expression. It has been demonstrated that PKR protein is decreased during EMCV infection^22^ and is degraded during infection with poliovirus^27^ or rift valley fever virus^28^. Activation of PKR is not required for its proteolysis since a catalytically inactive PKR is still degraded in poliovirus-infected cells^27^. Degradation of PKR during infections indicates ways by which viruses can surpass the inhibitory effect of PKR-eIF-2α system. We show here that EMCV enhanced PKR-SUMO1 modification without altering its protein level; in contrast, EMCV increased PKR-SUMO3 modification 2 h post-infection resulting in its caspase-mediated degradation at a later stage. EMCV also enhanced PMLIII isoform SUMOylation leading to its degradation occurring in a proteasome-dependent manner^25^. Together, these findings reveal mechanisms evolved by EMCV to antagonize the PKR and PML pathways. In addition, we show that early post-infection, EMCV enhanced global SUMO2/3 modification, therefore it will be interestingly in future studies to identify cellular proteins conjugated to SUMO upon EMCV infection.

A previous work showed that EMCV induced decrease of PKR protein in cells infected at an MOI higher than 10^22^. Here we report that at an MOI of 2, PKR level were not affected in HeLa-wt and HeLa-SUMO1 cells whereas PKR was degraded in HeLa-SUMO3 cells, suggesting that SUMO3 promoted EMCV-induced PKR degradation.

SUMO1 and SUMO2/3 modify both common and different substrates and several lines of evidence suggest that SUMO1 and SUMO2/3, which formed a different subfamily, may serve distinct functions^3^. Key differences between subfamilies include expression levels^29^, susceptibility to deSUMOylating enzymes^30^ and the ability to form SUMO chains^31^. Indeed, SUMO2/3, but not SUMO1, can form polychains that are recognized by the ubiquitin E3 ligase, RNF4, resulting in the ubiquitination of SUMO2/3 conjugated proteins and their proteasomal degradation upon various stimuli^7,32^.

Taken together, our results show that SUMO1 and SUMO3 exert differential effects on PKR activation and degradation. SUMO1 expression results in a gain of PKR activity by increasing its activation whereas SUMO3 abrogates its activation upon poly(I:C) transfection or viral infection. In addition, EMCV increases PKR conjugation to SUMO1 and SUMO3 but only SUMO3 expression promotes EMCV-induced PKR degradation. These results shed a new light on the differential effects of SUMO paralogs on PKR activation and stability.

## Materials and Methods

### Materials

Human recombinant IFNα2 was sourced from Schering (USA) and ginkgolic acid (GA), used at a concentration of 100 μM, was from Merck (USA). The proteasome inhibitor, epoxomicin, used at a concentration of 10 μM was from Merck Millipore (France) and the caspase inhibitor, zVAD-fmk, used at a concentration of 50 μM was from Promega (France). Rabbit anti-SUMO1 (Sc-9060), rabbit polyclonal anti-Actin (sc-1615) antibodies, rabbit anti-PKR (K17, sc-707), mouse monoclonal anti-PKR (K13, sc-136038), rabbit polyclonal anti-STAT1 (sc-345), mouse monoclonal anti-Hsp90 (4F10, sc-69703) and goat polyclonal anti-eIF-2α (K17, sc-30882) antibodies were from Santa-Cruz Biotechnology (USA). Rabbit anti-SUMO2/3 was from Invitrogen (Thermo Fischer, France), anti-phospho-PKR (Thr451) antibody from Merck Millipore (France), monoclonal anti-Ubc9 antibody from Abgent (USA), rabbit monoclonal anti-phospho-eIF-2α(Ser51) and rabbit anti-histone H3 antibodies from Cell Signaling (USA). The rabbit anti-EMCV antibodies were from Ann Palmenberg (Madison, USA) and the rabbit anti-VSV were from Danielle Blondel (Université Paris-Saclay, France). Secondary antibodies conjugated to Alexa Fluor were purchased from Molecular Probes (Thermo Fischer, France). Poly(I:C) and SUMO1 transfections were performed using Fugene 6 (Promega, France), siRNA targeting Ubc9 (ON-TARGETplus siRNA SMARTpool) was purchased from Dharmacon (GE Healthcare, France) and transfected into cells using HiperFect transfection reagent (Qiagen, France).

### Cells, viral stocks and infections

HeLa cells and L929 cells were grown at 37 °C in DMEM supplemented with 10% foetal calf serum. HeLa cells stably expressing His-SUMO1 or His-SUMO3 cells were used as previously described^7^. Stocks of VSV (Mudd-Summer strain, Indiana serotype) (10^9^ PFU/ml), and EMCV (10^8^ PFU/ml) were grown in L929 cells. HeLa-wt and HeLa-SUMO expressing cells were infected at the MOI indicated in the figure legends by adsorption in 1 ml medium. After 1 h, the medium containing virus was removed and replaced with medium containing 2% FCS, and the cells were incubated at 37 °C. The supernatants were saved for the indicated times and viral titers were determined on these cells by measuring the 50% tissue culture infective dose (TCID_50_).

### Immunofluorescence analysis

Cells grown on glass coverslips were fixed with cold acetone 10 min at −20 °C. Cells were then prepared for immunofluorescence staining using rabbit anti-PKR antibodies and the corresponding secondary antibody conjugated to Alexa Fluor (Molecular Probes, Thermo Fischer, France). Cells were mounted onto glass slides by using Immu-Mount (Shandon, Thermo Fischer, France) containing DAPI. Confocal laser microscopy was performed on a Zeiss LSM 710 microscope.

### Purification of His_6_-tagged SUMO conjugates

HeLa-wt, HeLa-SUMO1 and HeLa-SUMO3 cells (10^7^) were lysed in denaturating buffer A (6 M guanidinium-HCl, 0.1 M Na_2_HPO_4_/NaH_2_PO_4_, 0.01 M Tris-HCl pH 8.0, 5 mM imidazole and 10 mM β-mercaptoethanol). After sonication, the lysates were mixed with 50 μl of Ni-NTA-agarose beads (Qiagen, France) for 3 h at room temperature. The beads were successively washed with buffer B (0.1% triton X100; 8 M urea, 0.1 M Na_2_HPO_4_/NaH_2_PO_4_, 0.01 M Tris-HCl pH 6.3, 10 mM β-mercaptoethanol), and subsequently eluted with 200 mM imidazole in 0.15 M Tris-HCl pH 6.7, 30% glycerol and 0.72 M β-mercaptoethanol. The eluates were then analyzed by Western blotting.

### Western blot analysis

For total cell extracts, cells were washed in PBS, scraped into Laemmli buffer, and boiled for 5 min. For cell fractionation, cells were lysed in hypotonic buffer (10 mM Tris-HCl, pH 7.65, 1.5 mM MgCl_2_, 1 mM DTT, 20 mM *N*-ethylmaleimide, protease inhibitors) and centrifuged at 500 × g for 15 min. The supernatant constituted the cytoplasmic fraction and the pellet resuspended in Laemmli buffer constituted the nuclear fraction. Proteins of the different extracts were separated by SDS-PAGE as previously described^7^.

## Electronic supplementary material

Supplementary Information

## References

[1] Kerscher O, Felberbaum R, Hochstrasser M (2006). Modification of proteins by ubiquitin and ubiquitin-like proteins. Annu Rev Cell Dev Biol.

[2] Hay RT (2013). Decoding the SUMO signal. Biochem Soc Trans.

[3] Flotho A, Melchior F (2013). Sumoylation: a regulatory protein modification in health and disease. Annu Rev Biochem.

[4] Liang Y-C (2016). SUMO5, a Novel Poly-SUMO Isoform, Regulates PML Nuclear Bodies. Sci Rep.

[5] Bohren KM, Nadkarni V, Song JH, Gabbay KHD, Owerbach A (2004). M55V polymorphism in a novel SUMO gene (SUMO-4) differentially activates heat shock transcription factors and is associated with susceptibility to type I diabetes mellitus. J Biol Chem.

[6] Geoffroy M-C, Hay RT (2009). An additional role for SUMO in ubiquitin-mediated proteolysis. Nat Rev Mol Cell Biol.

[7] Maarifi G (2015). Small Ubiquitin-like Modifier Alters IFN Response. J Immunol.

[8] Hannoun Z, Maarifi G, Chelbi-Alix MK (2016). The implication of SUMO in intrinsic and innate immunity. Cytokine Growth Factor Rev.

[9] Brantis-de-Carvalho CE (2015). MxA interacts with and is modified by the SUMOylation machinery. Exp Cell Res.

[10] Maarifi G (2016). MxA Mediates SUMO-Induced Resistance to Vesicular Stomatitis Virus. J Virol.

[11] García MA (2006). Impact of protein kinase PKR in cell biology: from antiviral to antiproliferative action. Microbiol Mol Biol Rev MMBR.

[12] Sadler AJ, Williams BRG (2007). Structure and function of the protein kinase R. Curr Top Microbiol Immunol.

[13] Proud CG (1995). PKR: a new name and new roles. Trends Biochem. Sci..

[14] Hershey JW (1991). Translational control in mammalian cells. Annu Rev Biochem.

[15] García MA, Meurs EF, Esteban M (2007). The dsRNA protein kinase PKR: virus and cell control. Biochimie.

[16] Koromilas AE, Roy S, Barber G, Katze N, Sonenberg N (1992). Malignant transformation by a mutant of the IFN-inducible dsRNA-dependent protein kinase. Science.

[17] Meurs EF (1992). Constitutive expression of human double-stranded RNA-activated p68 kinase in murine cells mediates phosphorylation of eukaryotic initiation factor 2 and partial resistance to encephalomyocarditis virus growth. J Virol.

[18] I.W. Jeffrey S (1995). Nuclear localization of the interferon-inducible protein kinase PKR in human cells and transfected mouse cells. Exp Cell Res.

[19] Okumura F (2013). Activation of double-stranded RNA-activated protein kinase (PKR) by interferon-stimulated gene 15 (ISG15) modification down-regulates protein translation. J Biol Chem.

[20] de la Cruz-Herrera CF (2014). Activation of the double-stranded RNA-dependent protein kinase PKR by small ubiquitin-like modifier (SUMO). J Biol Chem.

[21] Fukuda I (2009). Ginkgolic acid inhibits protein SUMOylation by blocking formation of the E1-SUMO intermediate. Chem Biol.

[22] Hovanessian AG (1987). Rapid decrease in the levels of the double-stranded RNA-dependent protein kinase during virus infections. Virology.

[23] Ungureanu D (2003). PIAS proteins promote SUMO-1 conjugation to STAT1. Blood.

[24] Papon L (2009). The viral RNA recognition sensor RIG-I is degraded during encephalomyocarditis virus (EMCV) infection. Virology.

[25] El Mchichi B (2010). SUMOylation promotes PML degradation during encephalomyocarditis virus infection. J Virol.

[26] Hakki M, Marshall EE, De Niro KL, Geballe AP (2006). Binding and nuclear relocalization of protein kinase R by human cytomegalovirus TRS1. J Virol.

[27] Black TL, Barber GN, Katze MG (1993). Degradation of the interferon-induced 68,000-M(r) protein kinase by poliovirus requires RNA. J Virol.

[28] Habjan M (2009). Superti-Furga, H. Unger, F. Weber, NSs protein of rift valley fever virus induces the specific degradation of the double-stranded RNA-dependent protein kinase. J Virol.

[29] Saitoh H, Hinchey J (2000). Functional heterogeneity of small ubiquitin-related protein modifiers SUMO-1 versus SUMO-2/3. J Biol Chem.

[30] Kolli N (2010). Distribution and paralogue specificity of mammalian deSUMOylating enzymes. Biochem J.

[31] Tatham MH (2001). Polymeric chains of SUMO-2 and SUMO-3 are conjugated to protein substrates by SAE1/SAE2 and Ubc9. J Biol Chem.

[32] Tatham MH (2008). RNF4 is a poly-SUMO-specific E3 ubiquitin ligase required for arsenic-induced PML degradation. Nat Cell Biol.

