# Supplementary material for: Differential effects of SUMO1 and SUMO3 on PKR activation and stability
Source: Sci Rep. 2018 Jan 19;8:1277. doi: 10.1038/s41598-018-19683-6 (PMC5775435; doi:10.1038/s41598-018-19683-6)
Supplement: Supplementary file 1 — Supplementary Information [file 41598_2018_19683_MOESM1_ESM.pdf]

SREP-17-22419C

Differential effects of SUMO1 and SUMO3 on PKR activation and stability

Ghizlane Maarifi[1], Faten El Asmi[1], Mohamed Ali Maroui[1], Laurent Dianoux[1], Mounira K. Chelbi-Alix[1*]

[1]INSERM UMR-S 1124, Université Paris Descartes, 45 rue des Saints-Pères, 75006 Paris, France
[*]Corresponding author: mounira.chelbi-alix@parisdescartes.fr

Keywords: PKR, SUMO, VSV, EMCV, double stranded RNA

Supplementary figure legends:

Supplementary Figure 1 : Uncropped blot images in Figure 1

Supplementary figure 2 : Uncropped blot images in Figure 3

Supplementary figure 3 : Uncropped blot images in Figure 4

Supplementary figure 4 : Uncropped blot images in Figure 5

Supplementary figure 5 : Uncropped blot images in Figure 6

1b

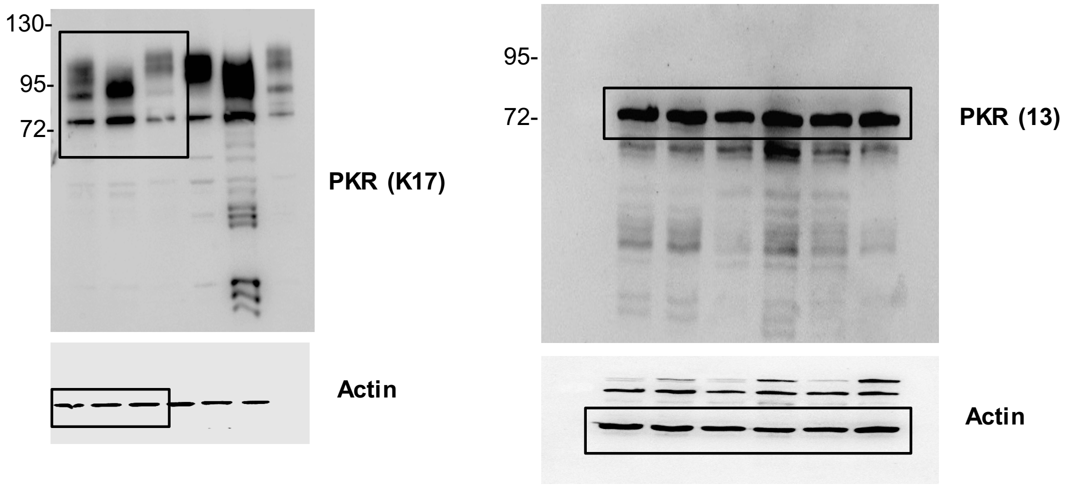

1c

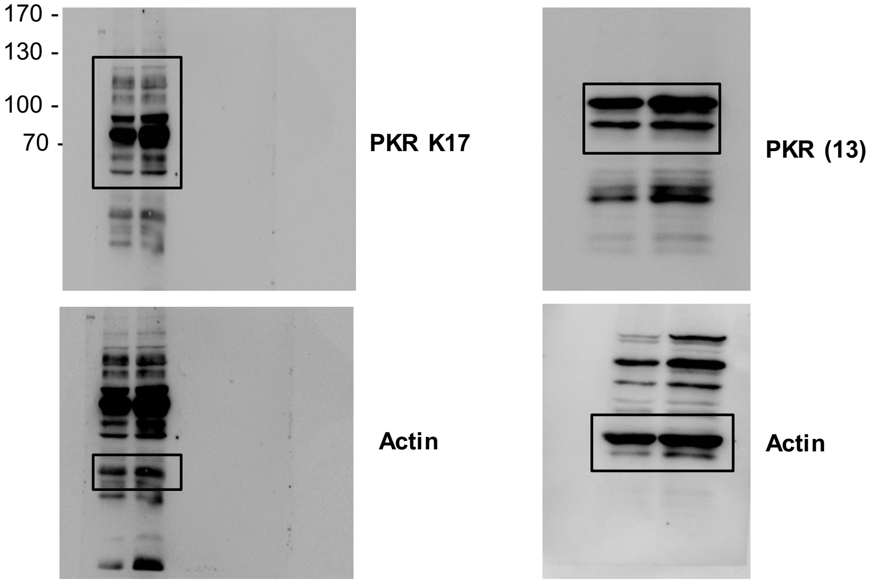

1d

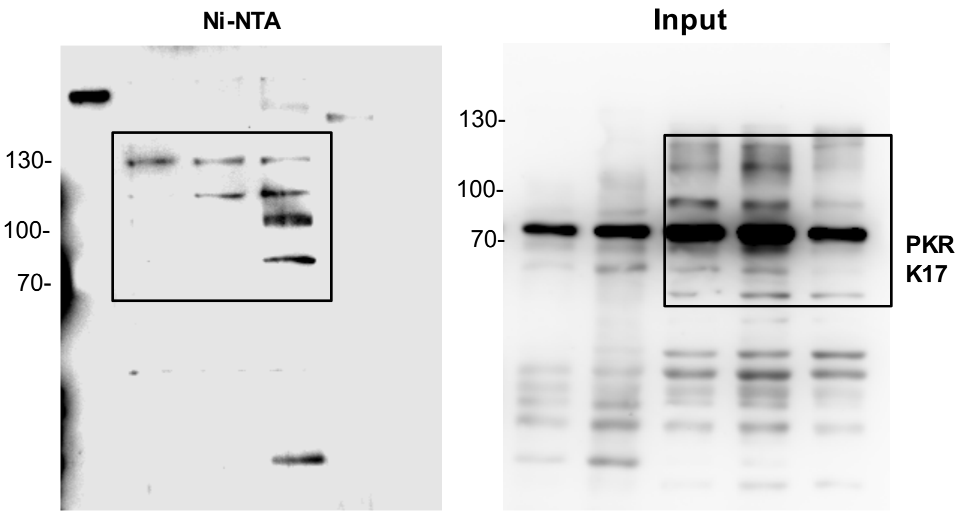

Supplementary Figure 1

3a left panel

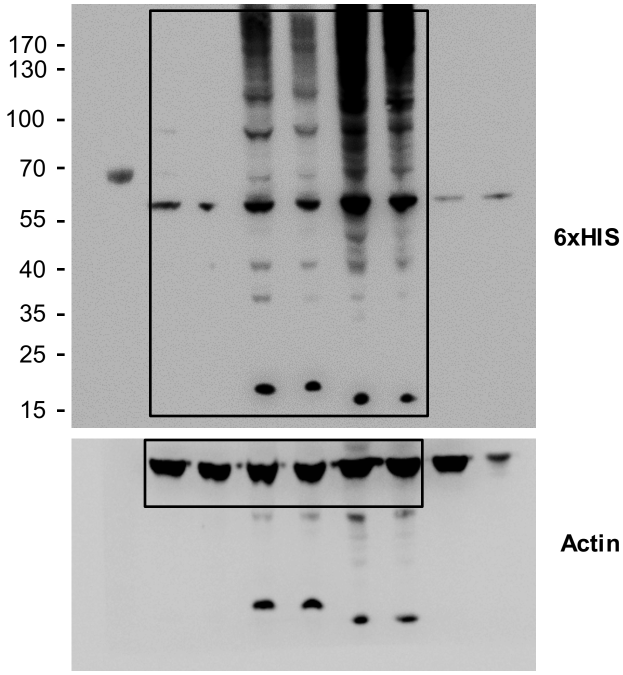

3a middle panel

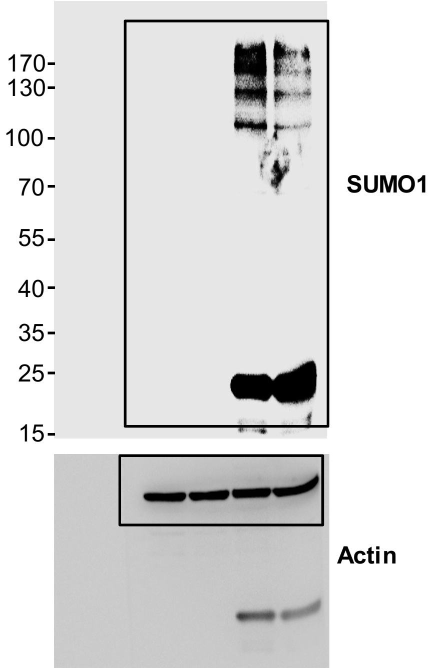

3a right panel

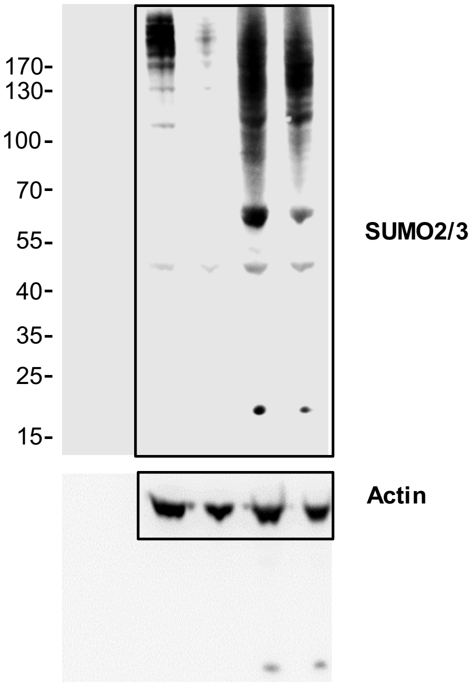

3b

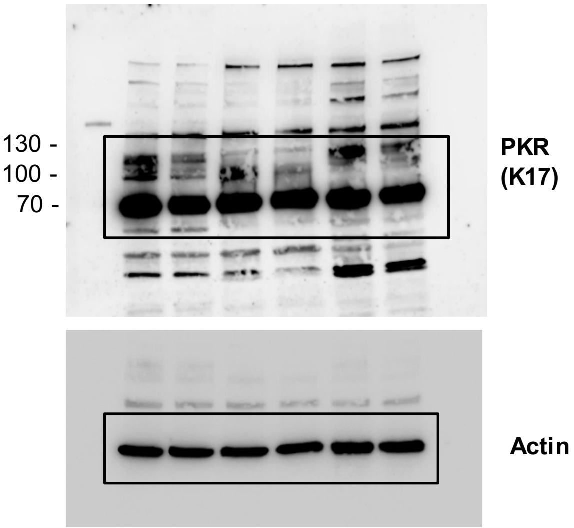

Supplementary Figure 2

3c left Panel

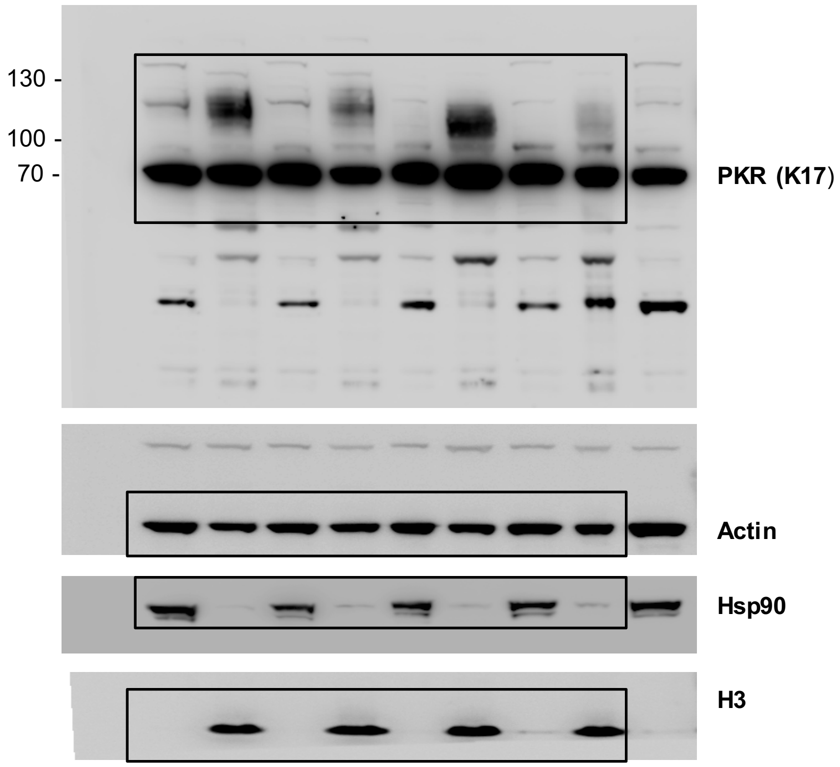

3c right panel

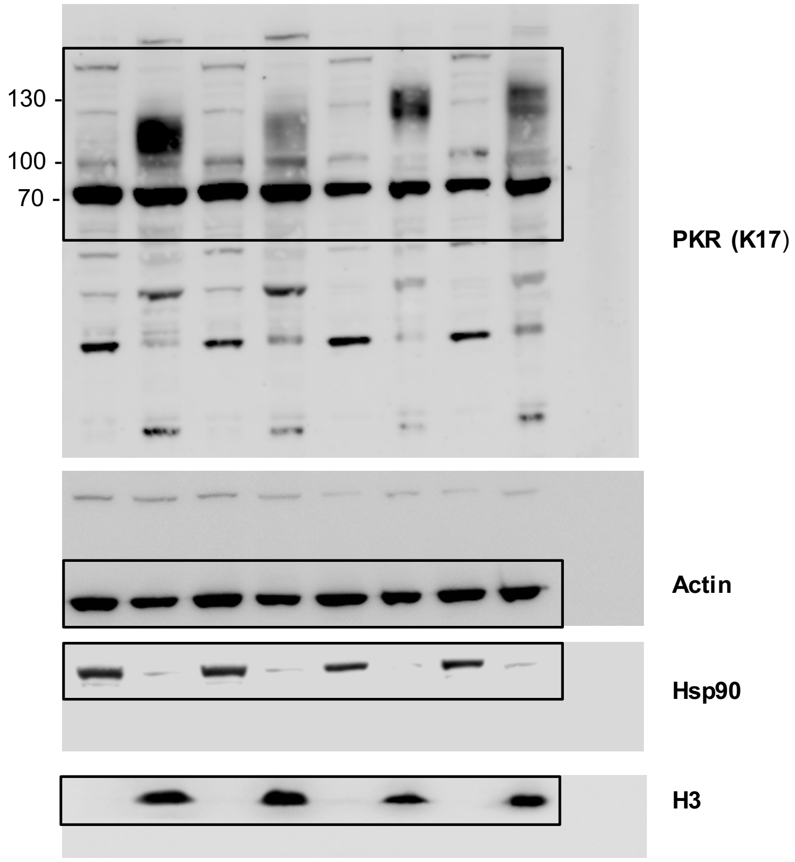

Supplementary Figure 2

4a

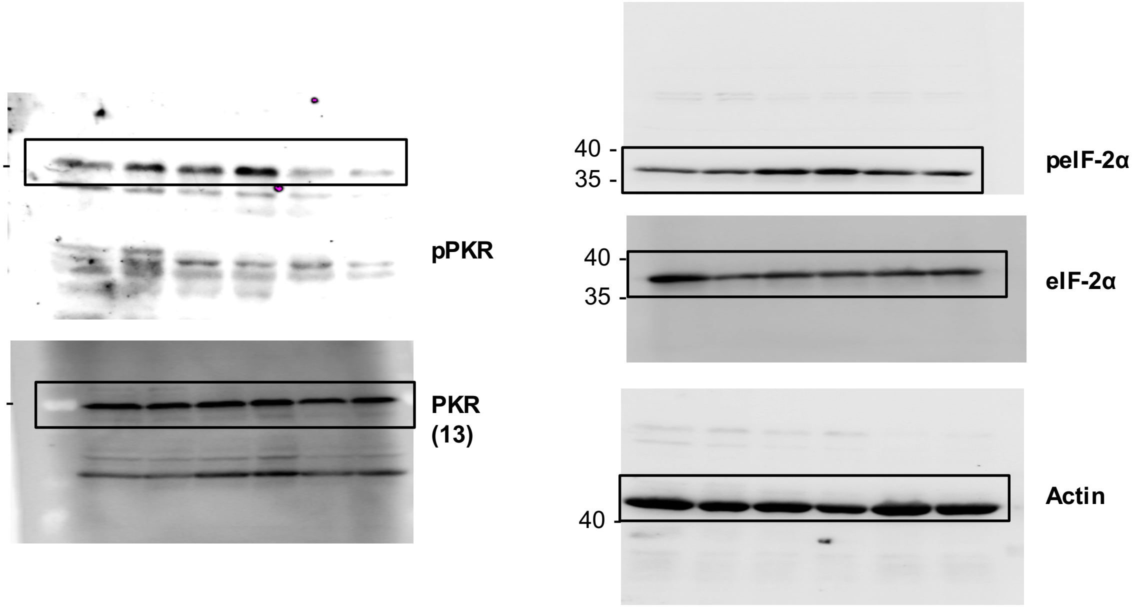

4b

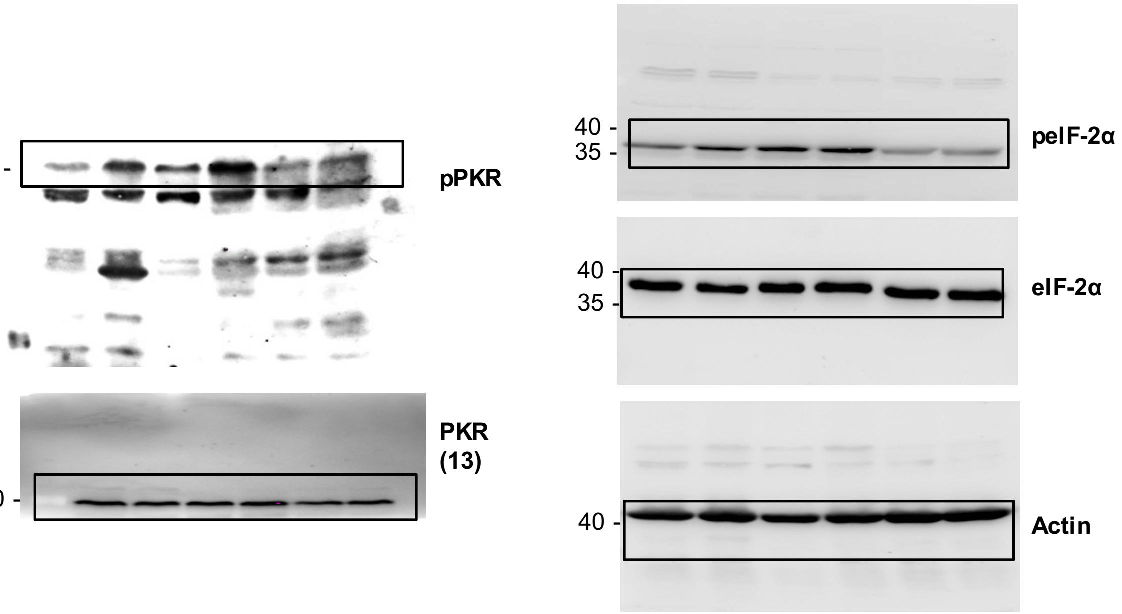

4c

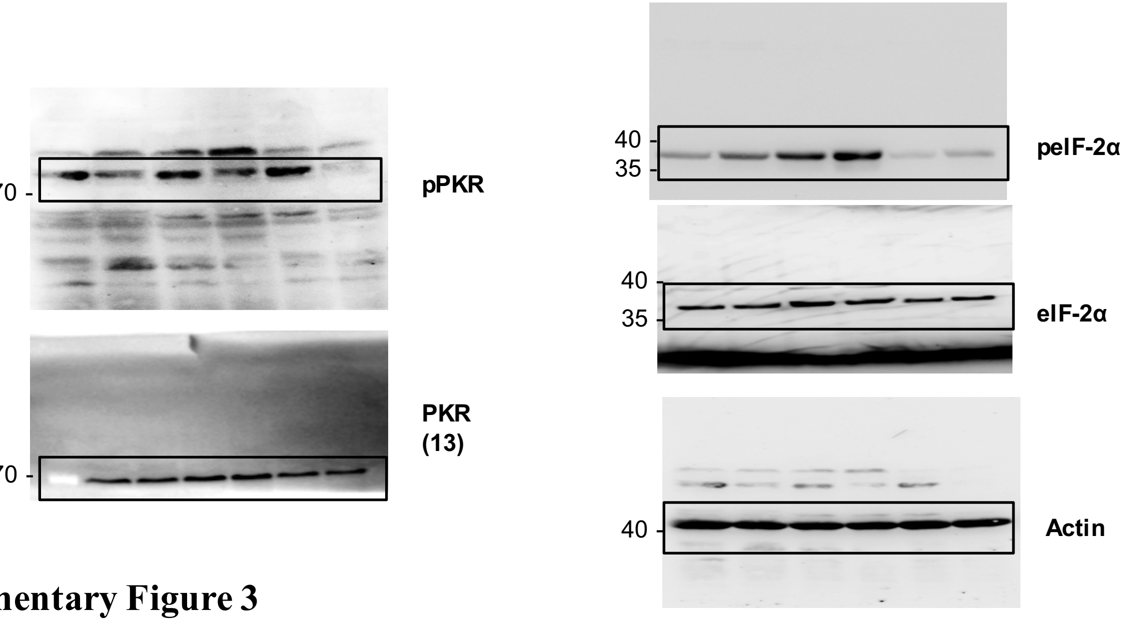

Supplementary Figure 3

4d left panel

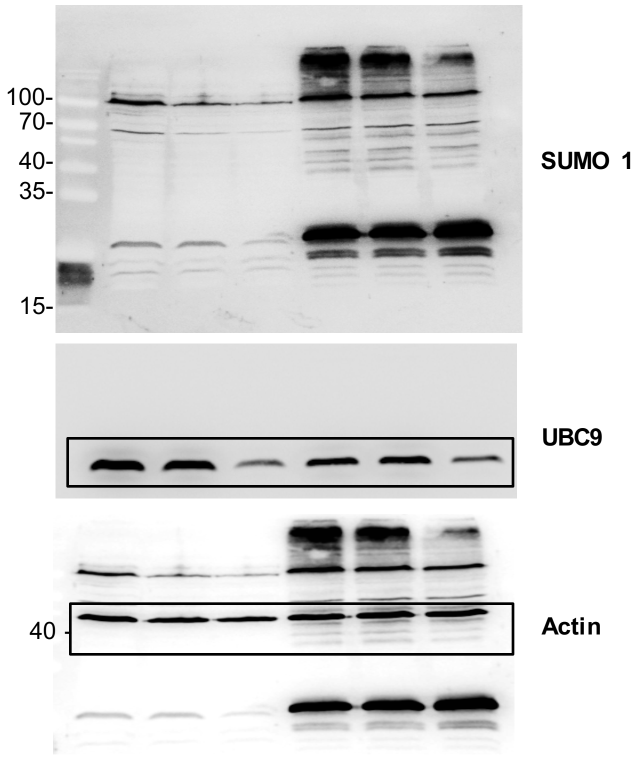

4d right panel

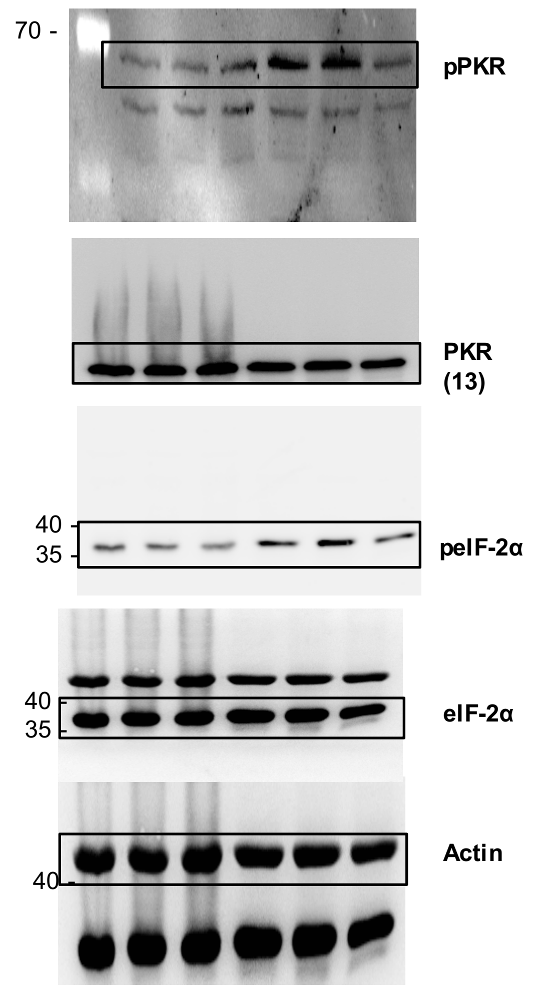

4e

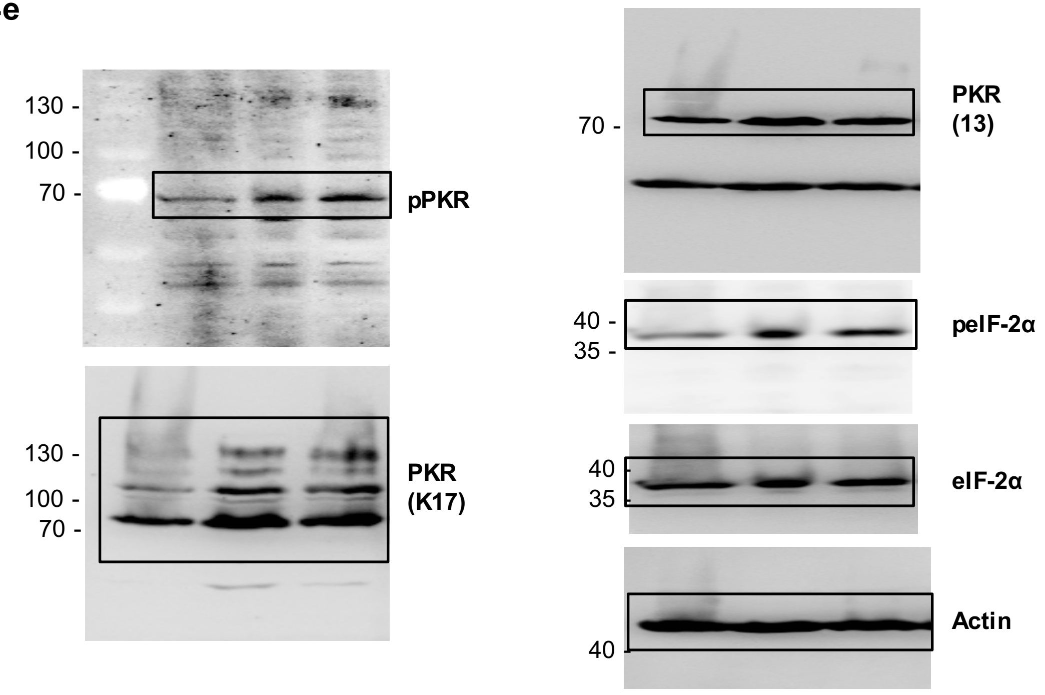

Supplementary Figure 3

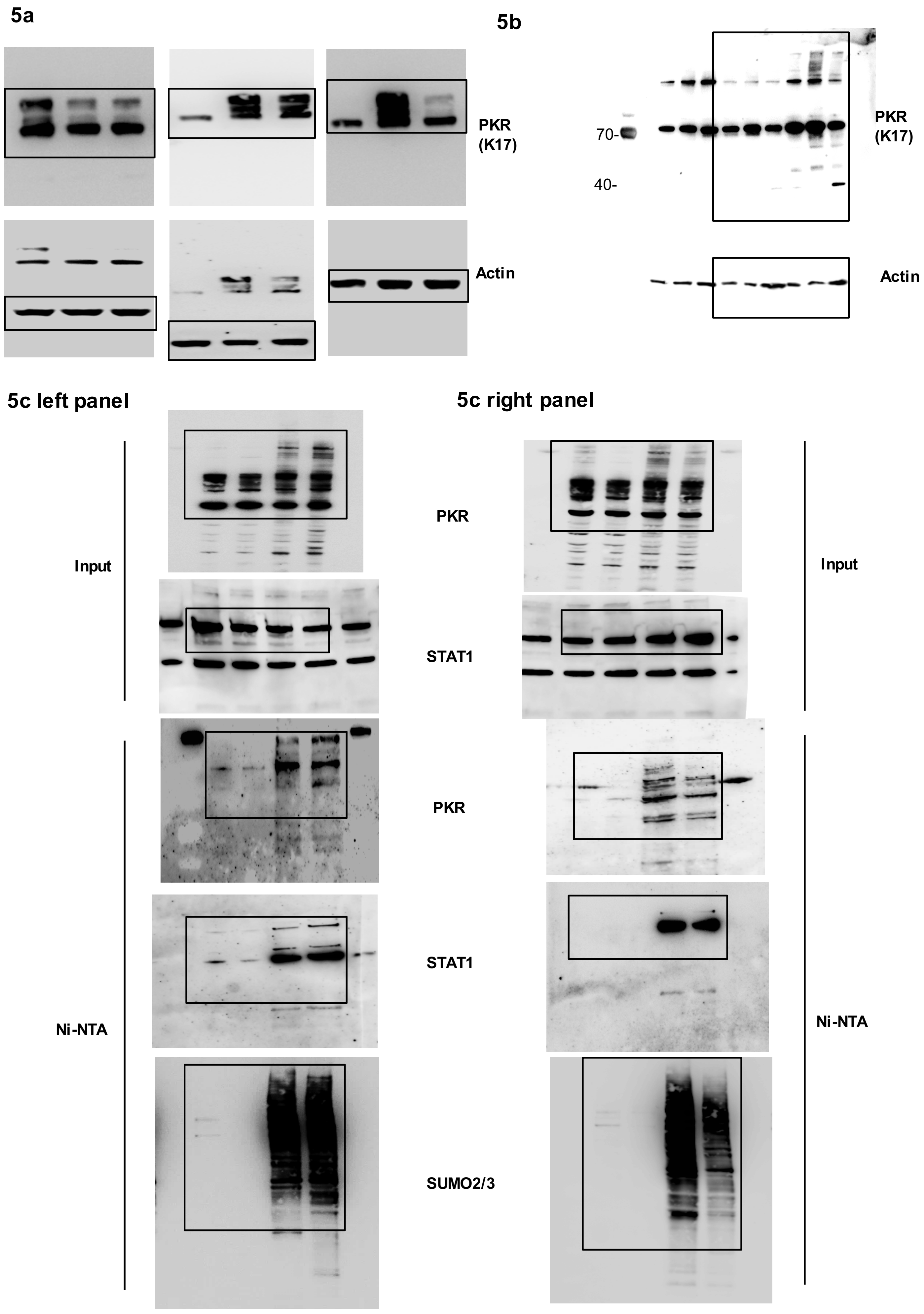

Supplementary Figure 4

5d left panel

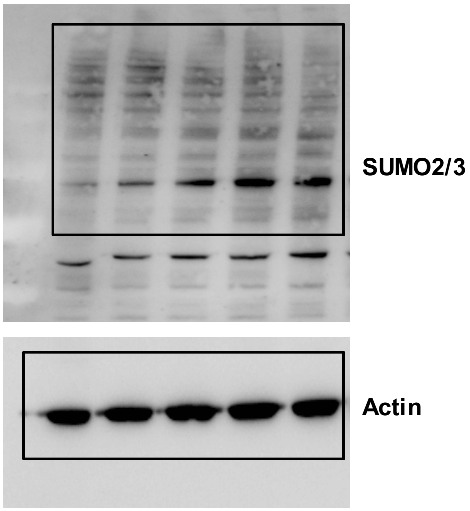

5d right panel

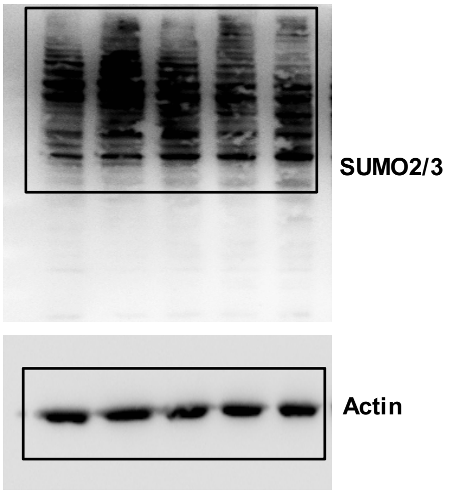

Supplementary Figure 4

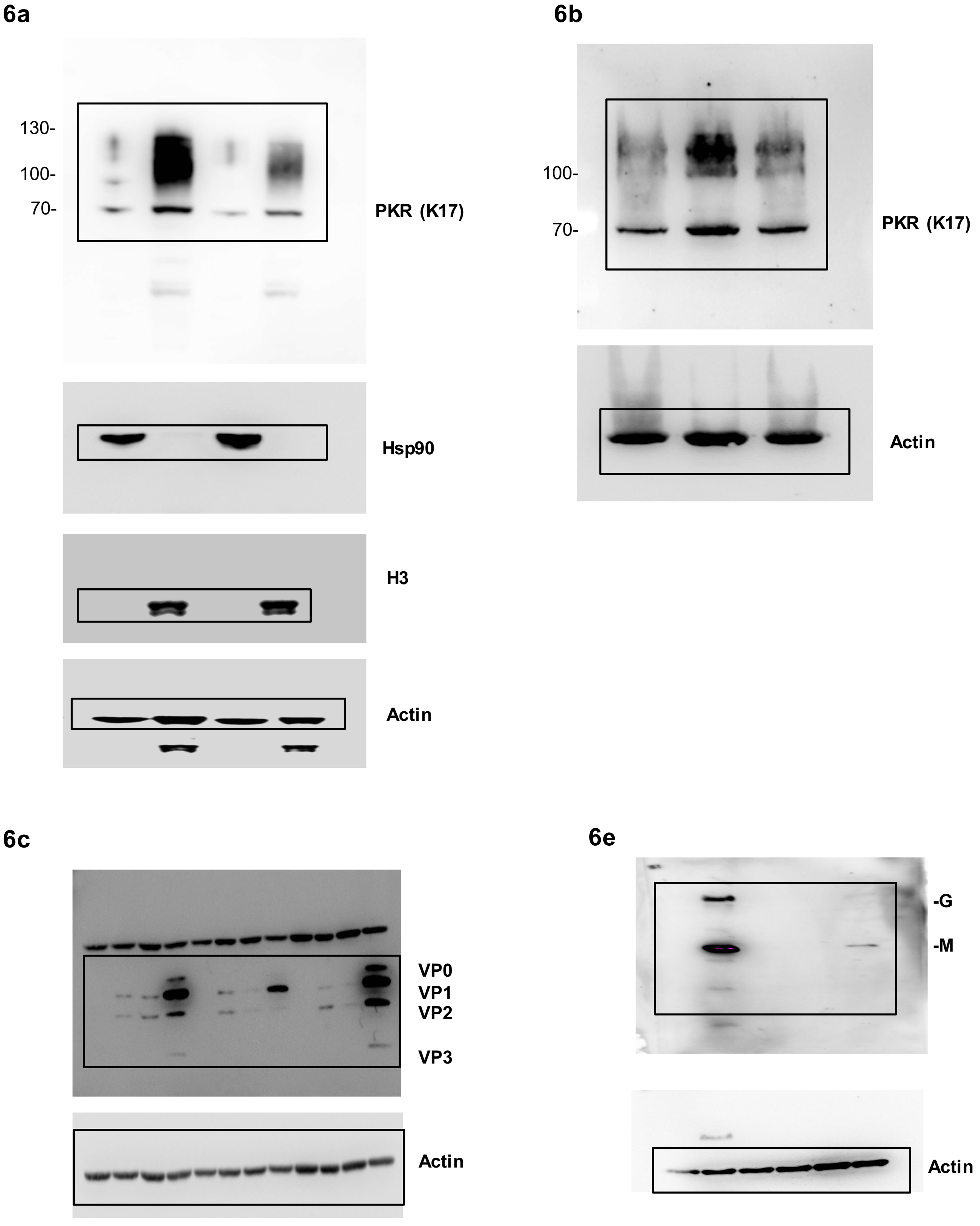

Supplementary Figure 5
